# Supplementary material for: One CGIAR and the Integrated Agri-food Systems Initiative: From short-termism to transformation of the world’s food systems
Source: PLoS One. 2021 Jun 4;16(6):e0252832. doi: 10.1371/journal.pone.0252832 (PMC8177634; doi:10.1371/journal.pone.0252832)
Supplement: S1 File — (DOCX) [file pone.0252832.s001.docx]

**Supplementary Information**

**Case study 1: Maíz para México – Development and validation of the IASI methodology**

In late 2016, a **window of opportunity** opened as internal changes at Mexico’s Ministry of Agriculture and external developments (particularly agricultural trade uncertainties) sparked interest in developing a national agricultural plan for the first time since 1966. The Ministry solicited support from CIMMYT with an initial focus on maize. **Analysis of current status** **(step 1)** began with aggregation of historical and contemporary data with the objective of developing a 2030 BAU scenario. (After eight months of assessing available data through an organic process that revealed many discrepancies across official national and international datasets and significant effort dedicated to aggregating municipal-level data, the decision was taken to use official national data to develop scenarios.) In parallel, CIMMYT convened an expert panel, composed of individuals with expertise in seeds, spatial analysis, climate change impacts on agriculture, farmer-market linkages, trade, pricing, and economic projections. CIMMYT provided the expert panel with a solutions-focused template to guide their recommendations for strategies to improve Mexican maize production by 2030. (Originally, an integrated recommendation was envisioned, but divergent views among panel members were accommodated, resulting in five proposed strategies.)

In March 2017, CIMMYT organized a **multi-sector consultation** **(step 2)** workshop that brought together eighty-five people from government, academia, and the agriculture sector. Using a non-traditional meeting space and carefully designed interactive processes, workshop discussions were focused toward a preferred 2030 scenario, drivers of change, and potential strategies. In parallel, the President of the National Agriculture Council (CNA), an influential private sector organization representing the agriculture industry as well as commercial farmers, announced that it would undertake a strategic planning process in which CIMMYT was invited to participate.

Throughout 2017, CIMMYT systematically reviewed inputs from the expert panel and the stakeholder workshop. Two main strategies emerged from this process, emphasizing commercial production and production for self-consumption. For each of these strategies, five drivers of change were defined along with a set of proposed actions with short-, medium- and long-term indicators. CIMMYT undertook a qualitative **validation (step 3)** process for each strategy, which included consultations with approximately twenty highly influential actors over a period of six months. These consultations yielded valuable additional insights and built commitment and ownership among policy decision makers and other influential actors. This resulted in a detailed report summarizing the process, findings, and **prioritized** strategies, which were socialized through consultations with other sectoral actors. In parallel, CIMMYT also led a strategic planning process for native maize seeds and production practices (i.e. the autochthonous, diversified, maize-based Milpa production system) as well as a Beans for Mexico initiative using the IASI methodology. By December 2017, the Mexican Agriculture Ministry released a strategic plan for thirty crops, based on a workshop replicating the design of the IASI exercise, that outlined time-bound targets and actions.

In the lead-up to the general election in July 2018, CIMMYT expanded the partnership space by briefing presidential candidates on the MpMx scenarios and stakeholder perspectives. With the election of a new president, greater political attention was directed toward national self-sufficiency in maize and other staple crops (e.g. beans, wheat, rice) and the government put forward goals for reducing consumption of processed food and encouraging consumption of domestic products. The incoming Minister of Agriculture was able to make links between the MpMx process and the food self-sufficiency initiative and frequently called on CIMMYT to inform policy discussions. In 2019, the **IASI strategic plan** **(step 4)** (Govaerts et al., 2019) was released and presented to the President’s office, the Minister of Agriculture, and the Minister of Economy. The President’s office made MpMx a flagship project within the new Crops for Mexico program and mandated CIMMYT to convene a taskforce with representation by two vice ministers and also to replicate the IASI methodology with beans and cotton (wheat and rice processes will be conducted under Crops for Mexico).

Through this taskforce, key public and private sector stakeholders worked toward a **shared strategy** **(step 5)** for achieving maize self-sufficiency in Mexico. Nine regions were identified by the taskforce where strategic governmental finance, research institutions, and MpMx-participating companies could collaborate on fostering local commercialization and building farmer capacities to increase yields through use of sustainable agricultural practices. During 2020, despite the restrictions of the COVID-19 pandemic, virtual events specified leaders and actions for MpMx implementation. Commitments by leading companies in the maize sector to buy specific volumes of maize directly from participating farmers represented an unprecedented level of sector collaboration.

Robust commitment by the Mexican president’s office has been key to strengthening collaboration within the agriculture sector, reinforcing the importance of engagement in MpMx as a way to align public policies with the Sustainable Development Goals. The MpMx **project-level dashboard (step 6)**, created by CIMMYT, enabled monitoring of action toward MpMx targets and has also provided information to Mexico’s 2030 SDG Agenda office.

Further details of the MpMx process can be found in *Maíz para México: Plan Estratégico 2030* (available at <https://repository.cimmyt.org/bitstream/handle/10883/20219/60937.pdf?sequence=4&isAllowed=y>), which includes historical trends and future projection for maize consumption, production, and national self-sufficiency as well as BAU scenarios, engines of change, and tactical plans.

**Case study 2: Maíz para Colombia – Adaptation and refinement of the IASI methodology**

In a post-conflict period, Colombia’s under-developed maize sector was threatened by imports under international trade, opening a **window of opportunity** for applying the IASI methodology. In February 2018, as part of an official visit with the Colombian Minister of Agriculture, CIMMYT leaders met with agriculture sector stakeholders and described the MpMx approach. Having completed climate change projections for Colombia, CIAT was presented as a lead in-country partner for developing a Maíz para Colombia initiative. Key elements were to include: (i) collaborative assessment of the state of the maize sector; (ii) review of a business-as-usual 2030 scenario (which indicated a mismatch between maize production and demand for different maize types as well as climate-induced shifts in maize-suitable production areas); and (iii) building broad agreement about where the maize sector should go.

To initiate the **analysis of current status (step 1)** for MpCo, CIAT gathered data and developed scenarios, while CIMMYT took the lead in convening an expert panel and socializing the IASI methodology among relevant stakeholders. The expert panel encompassed representatives from key Colombia-based institutions including FENALCE (National Federation of Cereal and Legume Growers), UPRA (the Agriculture Ministry’s Rural Planning Unit overseeing rural territorial planning), ANDI (the National Business Association of Colombia, with 1100 member companies across multiple economic sectors), Agrosavia (the Colombian Agricultural Research Corporation, a public body composed of non-profit, scientific, and technical organizations), and CIAT (CGIAR’s International Center for Tropical Agriculture) as well as the international HarvestPlus program (which promotes nutritionally enhanced varieties of staple food crops)*.*

In May 2018, CIMMYT and CIAT participated in a stakeholder workshop, organized by the Agricultural Rural Planning Unit*,* with participation by government, academia, and the agriculture sector. At this workshop, CIMMYT presented the MpMx strategic plan and shared initial progress and plans for using the IASI methodology to develop MpCo. Subsequently, meetings with the Ministry of Agriculture as well as detailed **stakeholder consultations** were held to gather feedback on initial MpCo analysis. During this period, the expert panel agreed upon six drivers of change and corresponding preliminary strategies. In July 2018, as a new President and Agriculture Minister came into office, the set of sectoral stakeholders engaged in MpCo shifted somewhat, but the Agricultural Rural Planning Unit staff stayed in place, providing continuity for the initiative.

In August 2018, CIMMYT and CIAT convened a 60-person, **multi-sectoral stakeholder workshop (step 2)** at the CIAT campus, where fifty maize experts reviewed each of the expert-defined drivers of change. This represented the first time in many years that the private sector participated actively in agricultural planning. CIMMYT systematically reviewed inputs from the workshop, revised the proposed MpCo strategies, and in November 2018 presented these to the Vice Minister for Agricultural Production and the agriculture committee of Colombia’s national congress. After receiving positive responses, CIMMYT undertook qualitative **validation** **and prioritization (step 3)** of the revised strategies through one-on-one meetings with leaders in government and the maize sector, leading to a **completed action plan (step 4)** in March 2019 that outlined MpCo 2030 **targets** and short-, medium-, and long-term **actions**. In June 2019, the IASI document on MpCo was publicly released (Galeano et al., 2019) and in early 2020, the National Agriculture Planning Office created an official plan based on this MpCo report. To reach an **expanded set of partners**, a CIMMYT-CIAT task force was given a mandate early in 2020 to seek resources for **implementation** of the validated strategies and potential collaborations were explored through one-on-one meetings with national and international stakeholders (e.g. FAO, the World Bank, the European Union).

Strategies and actions toward a preferred 2030 scenario for the maize sector, developed through the IASI-based MpCo, have become the **basis for planning (step 5)** by the Colombian government (the Maize Production Management Plan and Strategy) and FENALCE, with CIMMYT and CIAT serving as innovation brokers. An MpCo **project-level dashboard (step 6)** is being developed by CIMMYT and donor-funded projects that support elements of the MpCo have been initiated.

Further details of the MpCo process can be found in *Maize for Colombia: 2030 Vision* (available at https://repository.cimmyt.org/handle/10883/20382), which includes historical trends and future projection for maize consumption, production, and national self-sufficiency as well as BAU scenarios, drivers of change, and a strategic plan.

**Overview of data and analyses needed for application of the IASI methodology**

In application of the IASI methodology, four types of agri-food system data are gathered from existing databases:

- Historical agri-food system data including production, yield, area sown and harvested, demand, local and national consumption, and varieties most consumed.
- Environmental data including precipitation, temperature, solar radiation, and soil variables (e.g. texture, soil hydrological properties, rooting depth and fertility class).
- Crop characteristics including minimum and maximum values for growing cycle duration, crop temperature suitability, and crop rainfall requirements.
- Agronomic management data for crop model simulations including varieties used, sowing dates, sowing density, row distance, irrigation amount / frequency / depth, and fertilizer use.

Typical sources of data include: FAOSTAT; Climate Hazards Infra-Red Precipitation with Stations (CHIRPS); ERA5 agro-meteorological database; NASA-POWER; CHIRTS, SoiGrids, iSDA soil, and (sub-) national databases.

In application of the IASI methodology, current status (“where are we today?”) and the BAU trajectory (“where are we heading?”) are assessed through:

1. Review of literature and historical trends (i) to develop a comprehensive picture of production, processing, distribution, consumption, and trade patterns (i.e. geographical distribution, trend analyses, statistical centrality, and dispersion measures are computed at the national and subnational level, disaggregated by crop market segments); (ii) to characterize producer typologies by geography, scale of operation, and crop productivity; and (iii) to identify agri-food systems challenges (e.g. national self-sufficiency; food and nutritional security; climate vulnerability; value chain bottlenecks)*.*
2. Analysis of biophysical changes (i) to develop future (2030) projections of a crop’s climatic suitability under current and future climates, based on crop growing areas and productivity levels, and (ii) to identify current production areas where the crop becomes less suitable and new potential areas for production, based on optimal ranges of temperature and rainfall.
3. Socio-economic analysis (i) to develop future (2030) projections of socioeconomic impact on crop yield, production, and harvested area, in the context of climate change, and (ii) to explore how economic incentives (e.g., through international trade) could offset or exacerbate changes in biophysical crop fitness caused by climate change.

Models may include EcoCrop, DSSAT, APSIM, AquaCrop, SSM, GLAM, CropSyst, and IMPACT.
