# Supplementary material for: One CGIAR and the Integrated Agri-food Systems Initiative: From short-termism to transformation of the world’s food systems
Source: PLoS One. 2021 Jun 4;16(6):e0252832. doi: 10.1371/journal.pone.0252832 (PMC8177634; doi:10.1371/journal.pone.0252832)
Supplement: Attachment — Submitted filename: PONE-D-21-05705_Response to reviewers_DRAFT_28 Apr 2021.docx [file pone.0252832.s002.docx]

### Response to reviewers

| Reviewer comments | Response to reviewer comments |
| --- | --- |
| Reviewer #1: The manuscript **does not describe a technically sound piece of scientific research** (conducted rigorously, with appropriate controls, replication, and sample sizes) with **conclusions drawn appropriately based on the data presented**.  Reviewer #1: The authors have **not made all data underlying the findings in their manuscript fully available** (as part of the manuscript or its supporting information, or deposited to a public repository). | - As noted by the reviewer, this paper describes a decision support tool for agri-food systems (the IASI methodology) and presents the results of its application (a six-step, stakeholder-engaged process) in Mexico and Colombia. - These two country case studies constitute the research presented in this paper. The data underlying this study are qualitative, relating to the structured engagement, responses, and actions of agri-food system stakeholders. - The case studies are described methodically in the main manuscript (L170-257) and in greater detail in the Supplementary Information (IASI steps indicated with bolded text). - **To increase clarity, the six steps of the IASI methodology have been further highlighted by addition of parenthetical step numbers (matching the numbering in the Methodology text on L153-168).** - **New text has been added in the Methods section (L177) to more explicitly direct the reader to relevant public reports that expansively describe methods and findings.** - **New text has been added in the Supplementary Information (L71-75 and L131-134) indicating where readers can access further details related to historical trends and future projection for maize consumption, production, and national self-sufficiency as well as BAU scenarios, engines of change, and tactical plans.** |
| Reviewer #1: The manuscript has a large focus on this methodology **without a clear presentation of the results and the key messages of the study**.  Reviewer #1: The **conclusions and recommendations of this study is not clear**. It would be helpful for the readers when **conclusions and recommendations are presented separately**. | - Results can be found: in Table 1 and L187-257. - Conclusions can be found on: L31-34 (abstract); L284-293; L308-399. - Recommendations can be found on: L34-39 (abstract); L401-473 - **On L259, “Discussion” has been removed so the heading now reads “Insights from IASI development and validation”** - **The “Conclusions and recommendations” section is now called only “Recommendations.”** |
| Reviewer #1: The introduction section can further be strengthened by **highlighting the gaps in existing tools**. At the end of the introduction, the authors introduce IASI methodology is one of the decision support tools without introducing any other tools before.  Reviewer #1: The manuscript does not consist of **discussion in the context of existing studies or tools, highlighting the novelties of this new methodology**. | - **We agree that additional discussion is needed and have added new text (L99; L297-301) and citations (L555-557; L608-631) that enrich the attention to existing tools and challenges to their integration in policy development.** |
| Reviewer #1: The manuscript does not **present clearly the results of the applied IASI methodology**. Further, looking the methodology it is not clear **how decision makers can apply this methodology.** | - Application of the IASI methodology is described explicitly in the Methods section (L139-168). The steps of the IASI methodology are repeated in Table 1, which illustrates how the IASI methodology was applied in two country contexts. **To increase clarity, the steps in Table 1 have been numbered to match the numbering in the Methodology text.** - L188-199 reviews the “windows of opportunity” in Mexico and Columbia that enabled application of the IASI methodology. - L202-223 reviews how step 1 (current status / BAU scenario) and step 2 (preferred scenario / drivers of change) of the IASI methodology were applied in Mexico and Columbia. - L236-267 reviews key aspects of stakeholder engagement in Mexico and Columbia, which are central to steps 3-6 of the IASI methodology. - **A new section has been added to the Supplementary Information on “Overview of data and analyses needed for application of the IASI methodology” (L137-172).** |
| Reviewer #1: Please consider to **use terminologies such as tools, methodology, and framework in a consistent manner**. It would be better to use one term instead of multiple terminologies that say the same thing. | - “Tool” is used as a generic descriptor in the manuscript. For example, on L104-105: “As an example of one such tool, this paper describes the Integrated Agri-food Systems Initiative (IASI) methodology.” - “Methodology” is used when referring to IASI. - “Framework” is no longer used in reIation to the IASI methodology. It has been removed from L174. |
| Reviewer #1: L83-86: The main message of this statement is not clear. Do the authors referring to the existing synergies and trade-offs among SDGs? | - **The text has been modified for greater clarity.** |
| Reviewer #1: L141-143: Who are these stakeholders? Why “2030” is mentioned here? It can also be 2050 or other years. | - An indicative description of stakeholder types is provided on L80. **Text as has been added (L128-131) to clarify the types of stakeholders engaged in application of the IASI methodology.** - Scenarios and planning within the IASI methodology orient toward 2030 to be in alignment with the time frame of the UN Sustainable Development Goals. **This has been clarified in the manuscript on L207-208.** |
| Reviewer #1: L152-153: Does this dashboard need to be online? | - The IASI methodology is designed for application at the national (or large sub-national region) level, engaging diverse agri-food systems stakeholders, who are very commonly geographically dispersed, in a structured, transparent process. - It does not have to be an online dashboard, but an online dashboard, which can be accessed by diverse stakeholders at their convenience, is well-suited to broadening engagement beyond a core set of policy and sectoral ‘insiders.’ - In Table 1, the purpose of online dashboards is further explained (i.e. monitor progress toward the preferred scenario; support ongoing stakeholder engagement and course correction; to display investible opportunities for donors, government, and the private sector). |
| Reviewer #1: L184-186: Was that a systematic review? | - No, the IASI methodology does not include a formal systematic review. - L204-206 provides further detail on a component of Step 1 of the IASI methodology (as described on L153-155), specifically how “current status” is assessed based on relevant literatures and historical trend data. |
| Reviewer #1: L187-191: It is unclear what are these models. Are they crop models? | - Application of the IASI methodology is designed to adaptively integrate the best available data and models that are relevant to the agri-food system in question. - The IASI methodology is described on L126-1278 as a “multi-sector methodology that builds on diverse existing resources (e.g. datasets, models, approaches) to cultivate stakeholder agreement” that has its methodological origins in an Australian framework that relies “on loosely coupled biophysical, economic, and social models” (L120-121) to develop “model-based projections” for BAU and alternative scenarios (L153-156). - On L208-212, this description is provided: “Loosely coupled models (based on spatially-explicit analyses and machine learning processes) are used to produce sufficiently accurate and precise scenarios that use metrics and narratives to make risks, benefits, tradeoffs, and counter-intuitive insights visible.” - On L360-362, it is noted that future applications of the IASI methodology would benefit from “Deeper integration of system dynamics and climate change projections in scenario development and incorporation of flexible econometric and scenario planning models to generate a continuous range of options, rather than a limited number of scenarios.” - **New text has been added in the Methods section (L177) to more explicitly direct the reader to relevant public reports that expansively describe methods including models used to develop scenarios.** - **A new section has been added to the Supplementary Information on “Overview of data and analyses needed for application of the IASI methodology” (L137-172).** |
